# A biogeographical profile of the sand cockroach *Arenivaga floridensis* and its bearing on origin hypotheses for Florida scrub biota

**DOI:** 10.1002/ece3.3885

**Published:** 2018-05-15

**Authors:** Trip Lamb, Teresa C. Justice, Michael S. Brewer, Paul E. Moler, Heidi Hopkins, Jason E. Bond

**Affiliations:** ^1^ Department of Biology East Carolina University Greenville NC USA; ^2^ Lynchburg VA USA; ^3^ Florida Fish & Wildlife Conservation Commission Gainesville FL USA; ^4^ Department of Biology Ithaca College Ithaca NY USA; ^5^ Department of Biological Sciences and Auburn University Museum of Natural History Auburn University Auburn AL USA

**Keywords:** *Arenivaga*, dispersal, endemism, Florida platform, Gulf Coast corridor

## Abstract

Florida scrub is a xeric ecosystem associated with the peninsula's sand ridges, whose intermittent Pliocene–Pleistocene isolation is considered key to scrub endemism. One scrub origin hypothesis posits endemics were sourced by the Pliocene dispersal of arid‐adapted taxa from southwestern North America; a second invokes Pleistocene migration within eastern North America. Only one study to date has explicitly tested these competing hypotheses, supporting an eastern origin for certain scrub angiosperms. For further perspective, we conducted a genetic analysis of an endemic arthropod, the Florida sand cockroach (*Arenivaga floridensis*), with two aims: (1) to reconstruct the peninsular colonization and residence history of *A. floridensis* and (2) determine whether its biogeographic profile favors either origin hypothesis. We sequenced the *cox2* mitochondrial gene for 237 specimens (65 populations) as well as additional loci (*cox1*, nuclear *H3*) for a subset of Florida roaches and congeners. Using Network and Bayesian inference methods, we identified three major lineages whose genetic differentiation and phylogeographical structure correspond with late Pliocene peninsula insularization, indicating *Arenivaga* was present and broadly distributed in Florida at that time. Stem and crown divergence estimates (6.36 Ma; 2.78 Ma) between *A. floridensis* and western sister taxa span a period of extensive dispersal by western biota along an arid Gulf Coast corridor. These phylogeographical and phylogenetic results yield a biogeographic profile consistent with the western origin hypothesis. Moreover, age estimates for the roach's peninsular residence complement those of several other endemics, favoring a Pliocene (or earlier) inception of the scrub ecosystem. We argue that eastern versus western hypotheses are not mutually exclusive; rather, a composite history of colonization involving disparate biotas better explains the diverse endemism of Florida scrub.

## 1 INTRODUCTION

**Figure 1:**
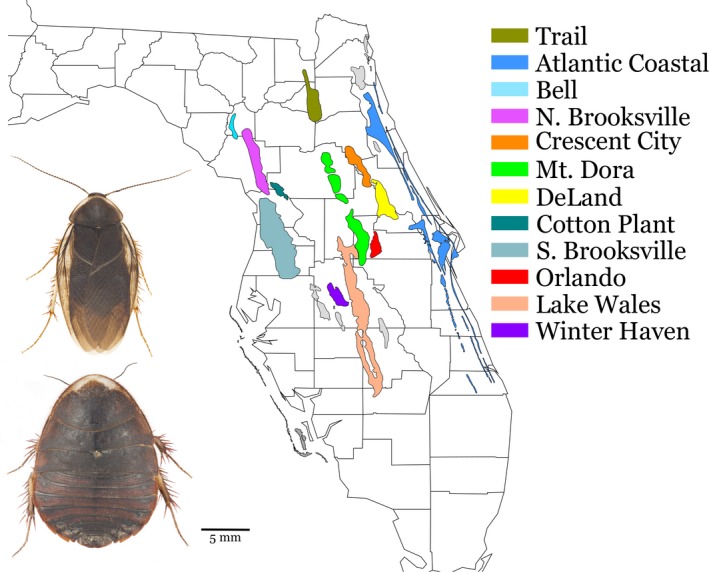
Map showing the Florida peninsula's sand ridge system, with sampled ridges depicted in color, and images of male and female (wingless) *Arenivaga floridensis*, illustrating species sexual dimorphism

Florida scrub is a fragmented xeric ecosystem largely confined to peninsular Florida (Menges, 1999), where it is partitioned across a series of relict beach ridges that formed sequentially during the Miocene, Pliocene, and Pleistocene epochs (Scott, 1997; Figure 1). Older ridges occupy the central peninsula, whereas the youngest corresponds roughly with present shorelines (Opdyke, Spangler, Smith, Jones, & Lindquist, 1984). Following inception, most ridges experienced rounds of inundation and isolation associated with sea level fluctuation (Webb, 1990). Today, these ridges retain scrub and related sandhill ecosystems, which were considered to have been widespread on the peninsula in the late Pleistocene (Myers, 1990) but experienced significant contraction to the ridges proper under more mesic conditions of the Holocene (Watts & Hansen, 1994).

Florida's sand ridges are characterized by quartzipsamment soils, providing porosity and drainage necessary to support the xeromorphic plant community that defines scrub—a shrubland composed of small evergreen oaks (*Quercus chapmanii* Sargent, *Q. geminata* Small, *Q. inopina* Ashe, *Q. myrtifolia* Willdenow) interspersed with Florida rosemary (*Ceratiola ericoides* Michaux) and maintained by low‐frequency, high‐intensity fires (Menges, 1999). Scrub is inherently patchy, spatially and temporally, and its historical flux, with attendant opportunities for isolation, is considered to have promoted speciation within this ecosystem. Indeed, Florida scrub is distinguished by high levels of endemism, which includes some 40 species of plants (Christman & Judd, 1990), four vertebrates (Moler, 1992; Rodgers, Kale, & Smith, 1996), and over 50 arthropods (Deyrup, 1989). Scrub endemics vary widely in overall distribution, with some species being confined locally within a single ridge and others occupying multiple ridges.

The origin of Florida scrub biota has been variously ascribed to Pliocene or Pleistocene epochs (Hubbell, 1961; Kurz, 1942; Neill, 1957), involving a “combination of both historical and edaphic factors” (Huck et al., 1989). One long‐held hypothesis invoking eastward range expansions of arid‐adapted biota into peninsular Florida (Myers, 1990) is based on a rich Pliocene fossil record representing numerous extralimital species with western or tropical affinities (Meylan, 1982; Morgan & Emslie, 2010). Western taxa purportedly dispersed along a recurrent Gulf Coast corridor—a broad belt of thorn scrub and savanna established by the increased aridity and lowered sea levels associated with Pliocene glaciation (Morgan & Emslie, 2010). Conversely, interglacial periods reduced corridor width, generated more mesic climatic conditions, and initiated Gulf Coast vicariance with regard to arid‐adapted species. Sundered from their western lineages, Florida populations underwent allopatric speciation in xeric peninsular settings. An alternative scrub origin hypothesis, detailed in Germain‐Aubrey et al. (2014), invokes southward dispersal events within eastern North America during Pleistocene glacial advances. Under this scenario, eastern taxa colonized the peninsula and, following local adaptation to xeric ridge environments, experienced ecological/spatial isolation sufficient for speciation (Swenson & Howard, 2005; Watts, 1975).

To test the two competing hypotheses, Germain‐Aubrey et al. (2014) generated molecular phylogenies for four angiosperm genera, focusing on the topological placement and sister taxon relationships of respective scrub endemics. Their results were ambiguous for one plant, the scrub plum (*Prunus geniculata* Harper), but they identified eastern origins for the remaining three species: Lewton's milkwort (*Polygala lewtonii* Small), scrub holly (*Ilex opaca* var. *arenicola* (Ashe) Ashe), and silk bay (*Persea humilis* Nash). However, age estimates for all four angiosperms dated to the Pliocene (or Miocene), a timeframe inconsistent with a Pleistocene colonization predicated by the eastern hypothesis. Additional reports have offered distributional data supporting eastern biogeographical sources for certain scrub taxa (Huck et al., 1989) and western sources for others (Hubbell, 1961; Zona & Judd, 1986), which suggests that eastern versus western origin hypotheses need not be mutually exclusive. With some 90 endemic species yet to be examined, a prevailing colonization pattern for Florida scrub has yet to be vetted.

Here, we examine the biogeography of a scrub arthropod, the Florida sand cockroach, *Arenivaga floridensis* Caudell (family Corydiidae). This species is the eastern representative of a genus otherwise distributed in arid settings from central Texas westward into California and Mexico (Hopkins, 2014a). As is characteristic of the genus, *A. floridensis* is fossorial and sexually dimorphic (females are wingless; Figure 1). Inhabiting scrub as well as adjacent sandhills communities, the species requires patches of open sand (Deyrup, 1994) and demonstrates a strong preference for loose substrate beneath light leaf litter of sand live oaks, *Quercus geminata* (Lamb, Justice, & Justice, 2006). Males occasionally engage in low, erratic flight at dusk, but juveniles and females appear to be completely fossorial (Deyrup, 1994). With populations documented from 11 peninsular ridges, *A. floridensis* ranks as Florida scrub's most geographically widespread faunal endemic (Lamb, Justice, & Justice, 2006).

The overall geographic distribution of *Arenivaga* appears consistent with expectations of the western origin hypothesis, underscoring the potential of *A. floridensis* to provide additional biogeographical perspective on scrub colonization and endemism. Support for the western origin hypothesis (with concomitant rejection of the eastern origin hypothesis) would require *A. floridensis* to meet the following predictions: (1) Pliocene colonization of the Florida peninsula, (2) a derived topological placement within a phylogeny of the genus (its basal placement could potentially negate a western origin of *Arenivaga*), and (3) a nodal divergence estimate for *A*. *floridensis* and its sister species approximating a late Miocene–early Pliocene timeframe. To distinguish between hypotheses, we present a detailed intraspecific phylogeography for *A. floridensis* in conjunction with a phylogeny for the species and selected congeners. We compare spatiotemporal patterns of our results with the aforementioned predictions to assess support or refutation of the western origin hypothesis.

## 2 METHODS

### 2.1 Sampling and sequencing regimens

To examine genetic variation in *Arenivaga floridensis,* we pursued a dense intra‐ and inter‐ridge sampling survey that yielded 237 roaches representing 65 localities throughout the species’ range (Appendix). Most specimens were captured by sifting sand samples through a two‐tier wire‐mesh (7.0 and 3.0 mm^2^) sieve, which retains all but the smallest nymphs. Roaches were preserved in 95% ethanol, and a rear leg of each was processed for genomic DNA using Qiagen's DNeasy kit. We selected the mitochondrial gene cytochrome oxidase II (*cox2*) as our initial genetic marker to assess population divergence and phylogeographical structure, using primers and amplification parameters listed in Table 1. Amplicons were cleaned using exoSAP‐IT (USB Corp.) prior to assay on an Applied Biosystems 3130 capillary sequencer. Resulting sequences, edited and assembled in SEQUENCER 4.9 (GeneCodes, Ann Arbor, MI), were aligned in CLUSTALX ver. 2.0 (Larkin et al., 2007) and translated to ensure correct reading frames.

**Table 1:** Primers and cycling conditions used for DNA amplification

Gene	Primer	Sequence (5′–3′)	Anneal. temp. (°C)	Cycles	Reference
*cox1*	C1‐J‐2183	CAA CAT TTA TTT TGA TTT TTT GG	50	32	Simon et al. (1994)
	Roach‐t‐Leucine^a^	TCC ATT GCA CTA ATC TGC CA			This study
*cox2*	TL2‐J‐3037	ATG GCA GAT TAG TGC AAT GG	50	32	Simon et al. (1994)
	TK‐N‐3785	GTT TAA GAG ACC AGT ACT TG			
*H3*	HexAF	ATG GCT ACC AAG CAG ACG GC	61.5	45	Ogden and Whiting (2003)
	HexAR	ATA TCC TTG GGC ATG ATG GTG AC			

a A version of Simon et al.'s (1994) TL2‐N‐3014, modified slightly for roaches.

To explore aspects of origin and dispersal, we also generated *cox2* sequences for five additional species of *Arenivaga*. One species, *Arenivaga erratica* Caudell, is the putative sister species to *A. floridensis*, whereas the remaining four, *Arenivaga tonkawa* Hebard, *Arenivaga gumperzae* Hopkins, *Arenivaga gaiophanes* Hopkins, and *Arenivaga bolianna* (Saussure), represent increasingly divergent clades within the genus (Hopkins & Giermakowski, 2014). We combined *cox2* data for the western species and 32 *A. floridensis* (representing all major and minor ridges in our sample) with sequence data for two additional loci, mitochondrial cytochrome oxidase I (*cox1*) and nuclear histone 3 (*H3*), amplified as detailed in Table 1. *Arenivaga erratica* served as the outgroup for the *cox2* dataset; *Eupolyphaga sinensis* (Walker) and *Ergaula capucina* (Brunner von Wattenwyl), representing additional corydiid genera, were outgroup taxa for multilocus analyses.

### 2.2 Phylogeographical and phylogenetic analysis

To examine phylogeographical structure in *A. floridensis*, we constructed *cox2* haplotype networks using the TCS algorithm (Clement, Posada, & Crandall, 2000) implemented in POPART (Leigh & Bryant, 2015). We also generated phylogenetic networks in SPLITSTREE v4 (Huson & Bryant, 2006) using the NeighborNet approach with default parameters. We used Bayesian inference (BI) analysis to estimate phylogenetic relationships within *A. floridensis* (*cox2* dataset) and, subsequently, among species (multilocus dataset). Best‐fit codon partitioning schemes and nucleotide substitution models were selected using the Bayesian information criterion (PartitionFinder v.1.1.0; Lanfear, Calcott, Ho, & Guindon, 2012). We used MRBAYES v.3.2.3 (Ronquist et al., 2012) to execute two concurrent runs involving four simultaneous chains of 20 million Markov chain Monte Carlo generations, sampling trees every 1,000 generations. The first 25% of the posterior distribution were discarded as burn‐in. Likelihood values for postanalysis trees and parameters were evaluated for convergence using the MRBAYES “sump” command and the program TRACER v. 1.6 (http://evolve.zoo.ox.ac.uk/software.html?id=tracer). We used the MRBAYES “sumt” command to generate a majority‐rule consensus tree and calculate posterior probabilities (PP) for consensus nodes.

### 2.3 Divergence time estimations

Divergence time estimates between the Florida lineages and between *A. floridensis* and western roach species were calculated using BEAST 1.8.2 (Drummond, Suchard, Xie, & Rambaut, 2012). Without fossil calibrations for *Arenivaga*, we used a substitution rate estimated across 15 beetle species for *cox2* (Pons, Ribera, Bertranpetit, & Balke, 2010) and an average of rates estimated for Hawaiian katydids (Shapiro, Strazanac, & Roderick, 2006) and beetles (Andújar, Serrano, & Gómez‐Zurita, 2012; Papadopoulou, Anastasiou, & Vogler, 2010; Pons et al., 2010) for *cox1*. Three partitions were analyzed (*cox1*,* cox2, and H3*; each separated by codon position) under PartitionFinder chosen substitution models and a birth–death speciation prior. Runs comprised 50 million generations and were sampled every 1,000 generations. To date the nodes, we used the following rates of substitution: *cox2 *=* *0.02610 substitutions per site per million yrs per lineage (subs/s/My/l) and *cox1 *=* *0.03605 subs/s/My/l. Under each model, the two nonfixed rates were estimated under a LogNormal relaxed clock. Two independent BEAST analyses were conducted and combined using LOGCOMBINER, with the first 10% of each run discarded as burn‐in. Convergence of parameters was accessed using TRACER, and trees were summed using TREEANNOTATOR.

In light of potential issues with concatenated gene data, we also produced a time‐calibrated species tree using StarBEAST2 (Ogilvie, Bouckaert, & Drummond, 2016) in BEAST2 v2.4.5 (Bouckaert et al., 2014). Site models, clock models, and topologies were unlinked between the mitochondrial and nuclear loci; ploidy assignments for *cox1* and *cox2* were designated haploid, and *H3* was designated diploid. The population model was set as “Analytical Population Size Integration.” Topologies and divergence times were estimated as in BEAST analysis above, using the *cox2* calibration rate.

## 3 RESULTS

### 3.1 Haplotype distribution, phylogeography, and phylogenetics

We obtained a 684‐bp fragment—effectively, the entire *cox2* gene—for all 237 specimens; sequence comparisons revealed 91 unique haplotypes (Appendix). Haplotype distribution was extremely localized, with 88 of the 91 haplotypes (97%) being observed at single localities. The three haplotypes (*Fp2*,* SB1*,* Vi*) representing more than one locality were limited to proximate sites 2–12 km apart. No haplotypes were shared between ridges despite the fact that certain inter‐ridge populations were within 5 km of each other. Such localized spatial distribution, in part a function of scrub patchiness, may also reflect the species’ limited vagility: *Arenivaga floridensis* is restricted to friable, sandy soils (Deyrup, 1994), and the wingless females may impose additional dispersal constraints.

**Figure 2:**
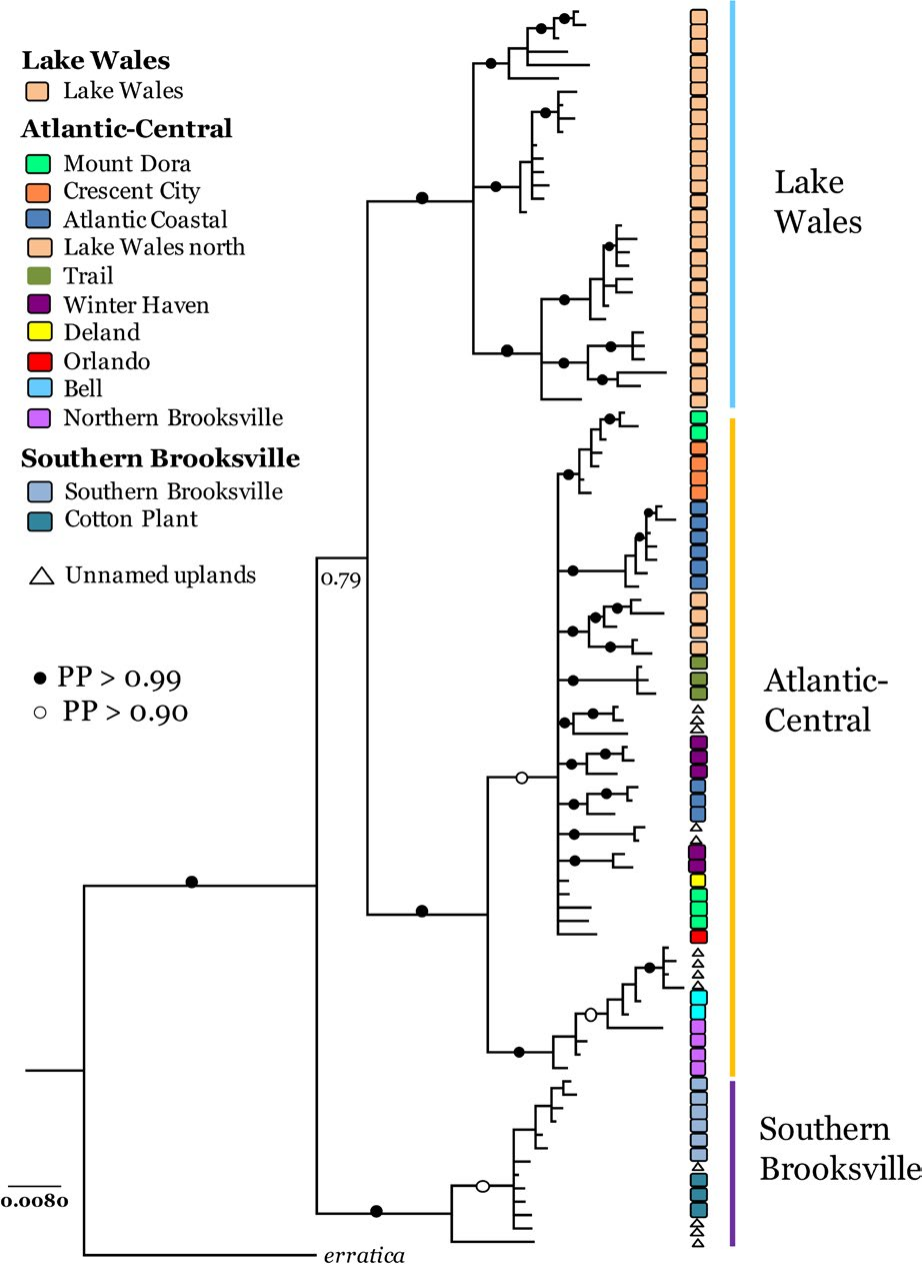
BI consensus tree from *cox2* dataset illustrating the three major lineages of *Arenivaga floridensis*, coded as: orange = Atlantic‐Central Lineage, blue = Lake Wales Lineage, and purple = Southern Brooksville Lineage (Figure S1 depicts *cox2* tree with labeled terminals and complete PP listings)

**Figure 3:**
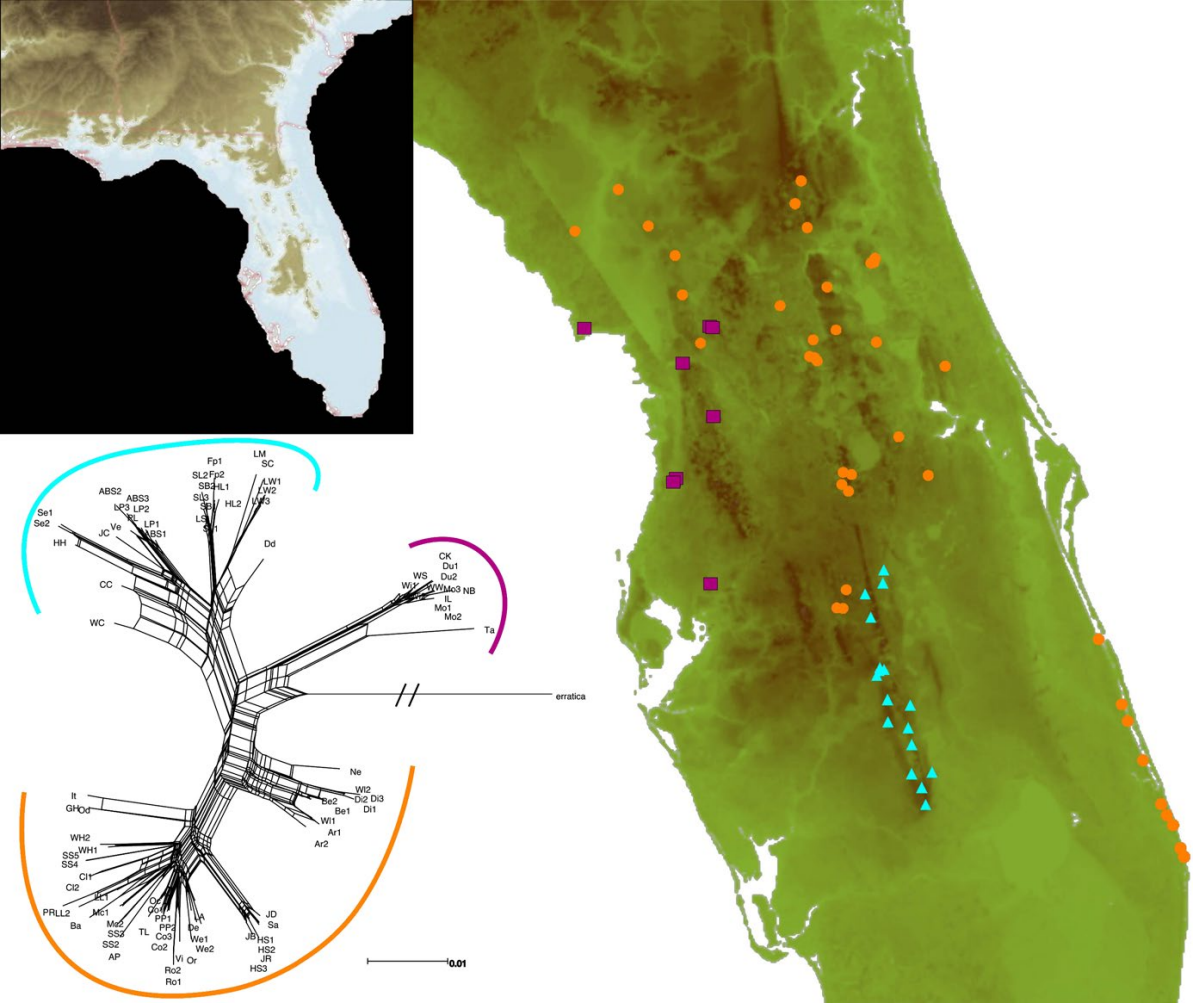
SPLITSTREE network and distribution map, illustrating the three major lineages of *Arenivaga floridensis*, coded as: orange = Atlantic‐Central, blue = Lake Wales, and purple = Southern Brooksville (see Appendix for haplotype abbreviations and localities). Inset map depicts peninsular uplands exposed during the Pliocene's last glacial minimum (3.2–2.7 Ma)

The BI consensus tree for *cox 2* identified three strongly supported lineages (PP > 0.99), with evident phylogeographical structure observed within and across lineages (Figures 2 and 3). The largest lineage, genetically and geographically, is designated the Atlantic‐Central Lineage. Comprising 50 haplotypes, it encompasses four major (Atlantic Coastal, Mount Dora, Northern Brooksville, and Trail) and five minor (Bell, Crescent City, DeLand, Orlando, and Winter Haven) ridges as well as adjacent sandy uplands. This lineage also includes a five‐member haplogroup (*Cl1*,* Cl2*,* LL1*,* LL2*, and *PR*) from the northern end of Lake Wales Ridge. However, the 18 remaining Lake Wales localities, which span the southern 140 km of the ridge's 186 km north–south axis, constitute a second distinct lineage of 29 haplotypes, appropriately termed the Lake Wales Lineage. The third lineage is distributed across a single major (Southern Brooksville) and minor ridge (Cotton Plant) and two disjunct scrub parcels (Cedar Key, Tampa Bay). With just 13 haplotypes, this, the Southern Brooksville Lineage, is the smallest but most genetically distinct lineage, constituting the sister group to the Atlantic‐Central/Lake Wales clade. These three major lineages were also identified in both SPLITSTREE (Figure 3) and TCS haplotype networks (not illustrated), the latter providing finer details of phylogeographic structure less pertinent to the predictions being tested.

**Figure 4:**
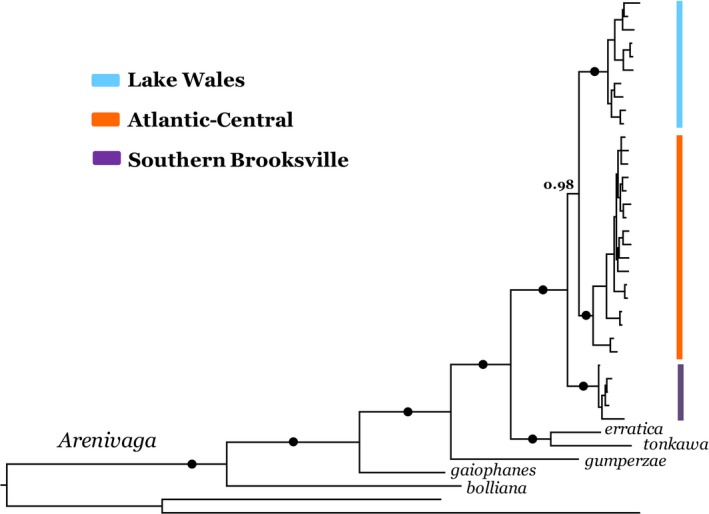
BI consensus tree from concatenated dataset for a subset of *Arenivaga floridensis* and selected western congeners. The three major lineages of *Arenivaga floridensis* are coded as: orange = Atlantic‐Central, blue = Lake Wales, and purple = Southern Brooksville. Circles indicate PP = 1.0; PP values for within‐lineage nodes are not shown, and outgroup terminals are not labeled. (Figure S2 depicts concatenated tree with labeled terminals and complete PP listings)

The three lineages were again recovered (all PP = 1.0) in the concatenated BI analysis but with higher nodal support for the Atlantic‐Central/Lake Wales clade (PP = 0.98; Figure 4) than in the *cox2* tree (PP = 0.79). The concatenated tree also reveals strongly supported interspecific relationships (all PP = 1.0), which are topologically congruent with Hopkins and Giermakowski's (2014) molecular–morphological phylogeny for the genus. Namely, *Arenivaga floridensis* is recovered as the sister taxon to an *A. erratica* + *A. tonkawa* clade, whereas the species *A. gumperzae*,* A. gaiophanes*, and *A. bolianna* represent increasingly divergent clades.

### 3.2 Divergence dating

**Figure 5:**
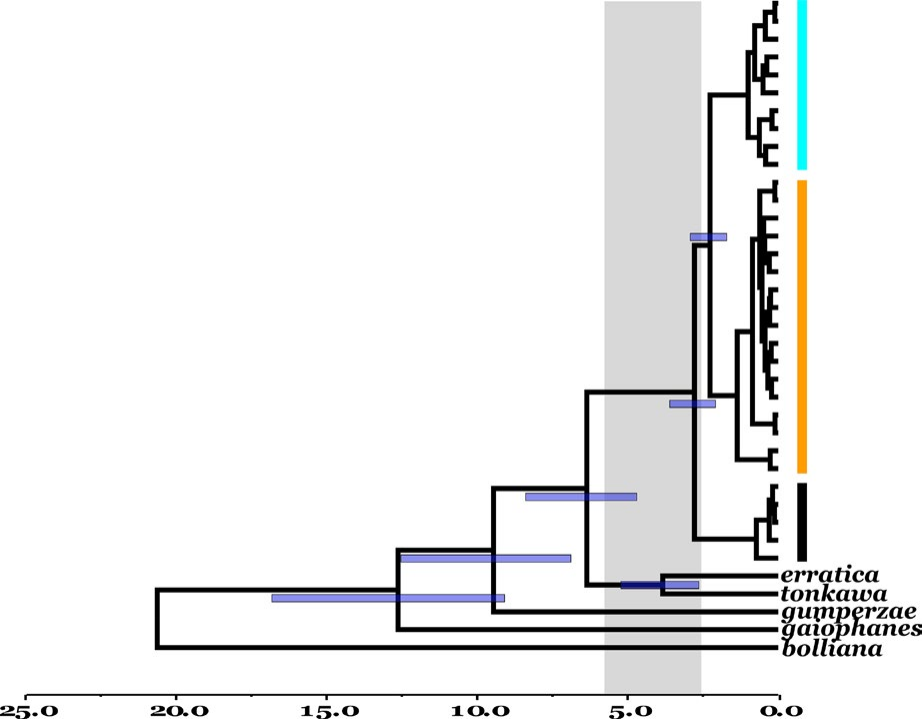
BEAST chronogram for the major lineages of *Arenivaga floridensis* and western *Arenivaga* for the concatenated dataset using *cox2* calibration. Blue HPD divergence bars subtend selected nodes, and the gray panel delimits the Pliocene (5.333–2.58 Ma). With exception of two nodes within lineages, support throughout the tree is PP >0.98; all HPD nodes have PP = 1. Lineages of *A. floridensis* are as follows: orange bar = Atlantic‐Central, blue = Lake Wales, and purple = Southern Brooksville

*Cox2* divergence time estimates within *A. floridensis* and among species are depicted in Figure 5. The nodal age between the Lake Wales and Atlantic–Central lineages is estimated to be 2.27 Ma (95% highest posterior density [HPD]: 2.92–1.71), whereas coalescence of all three roach lineages dates to 2.78 Ma (HPD: 3.61–2.08). The nodal age estimated for the *floridensis*–*erratica*+*tonkawa* clade is 6.36 Ma (HPD: 8.39–4.69). The species tree generated in StarBEAST2 yielded an identical topology (Figure S3), though with weaker nodal support (a function of limited variation in *H3*) and a slightly younger age estimate for *floridensis*–*erratica*+*tonkawa* clade (*cox2 *=* *4.02 Ma; HPD: 5.40–2.26). In turn, *cox1* divergence time estimates are older (BEAST consensus tree; Figure S4), with the Lake Wales and Atlantic–Central clade dating to 3.17 Ma (HPD: 3.98–2.47), lineage coalescence for *A*. *floridensis* to 3.88 Ma (HPD: 4.89–2.99) and the *floridensis*–*erratica*+*tonkawa* clade to 8.84 Ma (HPD: 11.33–6.61). Although the *Arenivaga* age estimates should be interpreted with caution in the absence of fossil calibration, we note that *cox2* and *cox1* HPD intervals exhibit substantial overlap for each of the three nodes. Furthermore, both genes offer general timeframes for peninsular colonization (late Miocene‐Pliocene) and ridge population isolation (Pliocene) that clearly predate the Pleistocene.

## 4 DISCUSSION

### 4.1 Phylogeographical structure and lineage ages

Our genetic survey of *Arenivaga floridensis* reveals a scrub endemic characterized by pronounced phylogeographical structure, much of it partitioned by sand ridges. Genetic differentiation among the three major lineages is likely attributable to isolation imposed by episodic marine transgressions, which inundated substantial portions of Florida during the Pliocene and Pleistocene epochs. Estimated lineage ages correspond closely with peninsular fragmentation during the Pliocene warm period (3.2–2.7 Ma) when sea levels rose significantly, with a peak some 22 m higher than present day (Miller et al., 2012). The specifics of the Florida platform's inundation require incorporating concomitant geologic factors such as isostatic uplift (Adams, Opdyke, & Jaeger, 2010; Rowley et al., 2013). Nonetheless, peninsular uplands emergent at this time were likely limited to portions of the Lake Wales, Mount Dora, Trail, and Southern Brooksville ridges, which offer striking geographical congruence with the Lake Wales, Central‐Atlantic, and Southern Brooksville lineages (Figure 3). The older age observed for the Southern Brooksville Lineage suggests a longer isolation history, which is consistent with the presence of specific Southern Brooksville endemics (Deyrup, 2005; Neill, 1957; Squitier, Deyrup, & Capinera, 1998) and notable absence of more widespread scrub species, for example, the Florida sand skink, *Plestiodon reynoldsi* (Stejneger), and Florida scrub lizard, *Sceloporus woodi* Stejneger. Uniquely, the Lake Wales Ridge is inhabited by populations representing two major lineages: the Lake Wales Lineage, present from the ridge's central portion to its southern terminus, and the Atlantic‐Central Lineage, represented by a 5‐member haplogroup confined to the ridge's northernmost sector. The presence of Atlantic‐Central haplotypes on the Lake Wales Ridge could reflect dispersal from the adjacent Mount Dora Ridge. However, phylogeographical structuring in a second scrub endemic, the aforementioned Florida sand skink, provides a more compelling interpretation. Substantive mtDNA divergence (*cob*, 4.5%) between skink populations from northern Lake Wales and those further south (Richmond, Reid, Ashton, & Zamudio, 2009) exhibits precise geographical congruence and a similar level of divergence (*cox2*, 5.5%) with roach haplotypes. Their genealogical concordance suggests a shared history whereby codistributed ancestral populations experienced vicariant separation in the northern Lake Wales Ridge. Overall, the phylogeography of *A. floridensis* reveals the combined influences of relatively early colonization (2.78 ma), close edaphic ties to peninsular ridges, and an episodic flux of scrub habitat under the climatic oscillations of Plio‐Pleistocene glaciation. Divergence levels detected for the three major lineages clearly support prediction 1 of the western origin hypothesis: *Arenivaga floridensis* has been present on the Florida peninsula since the Pliocene. Moreover, the roach's fidelity to Florida scrub and persistence through rounds of ecosystem expansion/fragmentation are aptly reflected in its phylogeographical complexity.

### 4.2 A western origin for *Arenivaga floridensis*

*Arenivaga* comprises a desert‐dwelling clade of roaches distributed largely within southwestern North America. The genus is now recognized as being far more species rich (48 vs. 9 spp) than traditionally perceived, with its highest levels of diversity centered in northern Mexico (Hopkins, 2014a). Although *A. floridensis* is considered to be the only species native to the southeastern United States, we note that two male specimens of *A. bolliana* have been collected in Florida (from the cities of Gainesville and Seminole). Otherwise, the eastern range boundary of *A. bolliana* terminates in Texas (some 1,400 km west); if viable populations do occur in Florida, they were probably recently introduced and remain extremely localized. Hopkins and Giermakowski's (2014) phylogeny of *Arenivaga* (24 of the 48 species) identified *A. erratica* as the sister species to *A. floridensis*, providing consistency with an earlier morphological interpretation that placed *A. floridensis* in the “*erratica* group” (Hebard, 1920). Our phylogeny differs slightly, recovering a sister group relationship between *A. floridensis* and *A. erratica* + *A. tonkawa*. This topological discrepancy probably reflects the influence of morphological characters in Hopkins and Giermakowski's (2014) integrated dataset. Hopkins’ (2014b) BI consensus trees for DNA sequence data, generated separately for *cox1*,* 12S,* and *H3* genes, depict slightly different relationships among these three species, although all are invariably recovered in the same clade. Topological placement of *A. floridensis*, relative to other congeners, reveals a derived position within the genus, both in our BI tree and in Hopkins and Giermakowski's (2014) phylogeny. As well, their phylogeny depicts a Baja California endemic*, Arenivaga diaphana* Hopkins, as the sister taxon to the remaining 23 species surveyed, which corroborates southwestern North America as the center of origin for *Arenivaga*. Our estimated dates for the most recent common ancestor of *A. floridensis* and *A. erratica* + *A. tonkawa* (6.36 Ma) provide a timeframe spanning the late Miocene to early Pliocene epochs. Arid conditions linking Florida and southwestern North America at this time facilitated biotic interchange and account for a strong western influence on the peninsula's Plio‐Pleistocene faunas (Krysko, Nuñez, Lippi, Smith, & Granatosky, 2016; Morgan & Emslie, 2010). Complementary patterns in diversity, distribution, and phylogenetic relationships for *Arenivaga*, together with nodal estimates for the *erratica*‐*floridensis*‐*tonkawa* clade*,* provide spatiotemporal consistency with an eastward migration from southwestern North America. These results satisfy predictions 2 (derived topological placement) and 3 (appropriate sister taxon divergence estimate) of the western origin hypothesis.

### 4.3 Reevaluating scrub origin hypotheses

Our phylogeographical and phylogenetic results for *Arenivaga* support a different scrub origin hypothesis than Germain‐Aubrey et al.'s (2014) angiosperm phylogenies but, importantly, indicate that the two hypotheses are not mutually exclusive. Although no other studies have actually tested either hypothesis, it is possible to infer eastern versus western origins for additional scrub endemics (or near endemics) from published phylogenies. For example, the evolutionary history of the Florida scrub‐jay exhibits remarkable parallels with that of *Arenivaga floridensis*. The Florida scrub‐jay—also the sole eastern representative of its genus—is sister taxon to four western species that form the ‘scrub‐jay group’ within *Aphelocoma* jays (McCormack et al., 2011). Sequence divergence (mitochondrial *cob* and *nad2*) between the Florida and western scrub‐jays, at 8.0%, with a nodal estimate of ~6.0 Ma (McCormack et al., 2011), corresponds with our observed *cox2* divergence and age estimate between *Arenivaga floridensis* and *A. erratica* + *A. tonkawa* (8.6%, 6.36 Ma). This case, together with phylogenies for additional taxa (Table 2), reveal unequivocal eastern and western contributions to Florida scrub biota. The eastern origin hypothesis advocates a Pleistocene inception of Florida scrub, invoking dispersal of eastern North American species onto the peninsula in response to glacial advances between 2.56 Ma and 10 ka. Compiling Germain‐Aubrey et al.'s (2014) phylogenetic results with those of others, we identified eight scrub endemics with sister species from eastern North America (Table 2). However, only two cases appear indicative of Pleistocene speciation. The first involves silk bay, *Persea humilis*, and closely related taxa, *Persea borbonia* (L.) Sprengel and *Persea palustris* (Rafinesque) Sargent, which form a clade dating to near the Pliocene–Pleistocene interface (Germain‐Aubrey et al., 2014). The second example involves the grasshopper *Schistocerca ceratiola* Hubbell and Walker, an obligate, sedentary specialist on Florida rosemary that exhibits minimal genetic distance across ridges (0.16%–0.3%; Lamb & Justice, 2005) and recent divergence (~0.6 Ma) from its sister species (Song, Moulton, Hiatt, & Whiting, 2013). If the western origin cases are also considered, then age estimates for nine of ten scrub endemics date to peninsular residence times of Pliocene age or older. Although Pleistocene glaciation unquestionably influenced intraspecific phylogeography in eastern North America (Avise, 2000; Soltis et al., 2006), its role in scrub speciation appears to have been minimal. Germain‐Aubrey et al. (2014) noted a Miocene cooling trend in eastern North American (Foster, Lunt, & Parrish, 2010) that may have elicited earlier southward migrations, which would provide greater temporal consistency with observed genetic differentiation between scrub endemics and their sister taxa. We have provided the first molecular phylogenetic evidence used to test and confirm the western origin hypothesis for a Florida scrub endemic and have compiled additional molecular phylogenies identifying both eastern and western progenitors to scrub species. These cases reveal more examples of eastern origin than western, and proximity alone would seem to favor eastern contribution potential over intermittent dispersal from the southwest. Still, we hesitate to endorse a prevailing eastern assembly when the evolutionary histories for most scrub species have yet to be determined. It is clear now, however, that arguments for a single regional source leading to the inception of this ecosystem can be rightly dismissed; rather, a composite biogeographical history involving disparate biotas better explains Florida scrub origins and endemism.

**Table 2:** Geographic origins and epoch age assignments for selected Florida scrub endemics

Taxon	Hypothesis support	Sister taxon range^a^	Epoch age assignment	Reference
Plants				
Florida rosemary (*Ceratiola ericoides)*	Eastern	NE	Miocene	Li, Alexander, Ward, Del Tredici, and Nicholson (2002) and Trapnell, Schmidt, Quintana‐Ascencio, and Hamrick (2007)
Scrub holly (*Ilex opaca* var. *arenicola*)	Eastern	E	Pliocene	Germain‐Aubrey et al. (2014)
Lewton's polygala (*Polygala lewtonii*)	Eastern	E	Miocene	Germain‐Aubrey et al. (2014)
Silk bay (*Persea humilis*)	Eastern	SE	Pleistocene	Germain‐Aubrey et al. (2014)
Scrub plum (*Prunus geniculata*)	Ambiguous	NA	Mio‐Pliocene interface	Germain‐Aubrey et al. (2014)
Scrub hickory (*Carya floridana*)	Eastern	SE	Pliocene	Zhang et al. (2013)
Florida jujube (*Condalia celata*)	Western	SW	_	Islam and Guralnick (2015)
Animals				
Florida scrub‐jay (*Aphelocoma coerulescens*)	Western	SW	Mio‐Pliocene interface	McCormack, Heled, Delaney, Peterson, and Knowles (2011)
Florida sand skink (*Plestiodon reynoldsi*)	Eastern	SE	Miocene	Brandley et al. (2011)
Florida scrub lizard (*Sceloporus woodi*)	Eastern	SE	_	Wiens, Kuczynski, Arif, and Reeder (2010)
Rosemary grasshopper (*Schistocerca ceratiola*)	Eastern	SE	Pleistocene	Song (2004) and Song et al. (2013)

a North American sectors abbreviated as: E = eastern, NA = North America, NE = northeastern, SE = southeastern, SW = southwestern.

## CONFLICT OF INTEREST

None declared.

## AUTHOR CONTRIBUTIONS

TL conceived the project; TJ, PM, TL, and JB collected specimens; TJ and TL generated/analyzed sequence data; MB and JB conducted network and phylogenetic analyses; HH provided material and sequence data for the western *Arenivaga*; TL wrote the manuscript; MB generated figures and contributed to the text.

## DATA ACCESSIBILITY

DNA sequences: GenBank accession numbers—DQ873966.1, DQ874280.1, FJ830540.1, KP986405.1, MG761831–MG761994.

## APPENDIX 1

### Localities, coordinates, sample sizes, and *cox2* haplotype designations (derived from locality abbreviations) for populations of *Arenivaga floridensis*, partitioned by ridges, from north to south

**Table:**

Ridge	Coordinates	*n*	Haplotype (n)
Trail		8	3
Gold Head State Park	29.82782, −81.94990	4	*GH* (4)
Ordway Preserve	29.72840, −81.97990	2	*Od* (2)
Interlachen	29.6243, −81.9198	2	*It* (2)
Atlantic Coastal		35	10
Roseland	27.82842, −80.47814	2	*Ro1* (1), *Ro2* (1)
Viking	27.54285, −80.36295	3	*Vi (3)*
Ft. Pierce	27.47035, −80.33381	5	*Vi* (5)
Savannas State Preserve	27.29906, −80.25837	4	*Sa* (4)
Hobe Sound A	27.10840, −80.16827	3	*HS1* (3)
Hobe Sound B	27.05972, −80.14045	5	*HS2*(4), *HS3* (1)
Jonathan Dickinson State Park	27.01693, −80.11015	3	*JD* (3)
Jupiter Ridge Natural Area	26.91608, −80.07305	4	*JR* (4)
Juno Beach	26.87847, −80.05540	6	*JB* (6)
Bell		4	2
Bell	29.79037, −82.85362		*Be1*(3), *Be2* (1)
Northern Brooksville		15	6
Archer	29.50267, −82.57250	5	*Ar1* (2), *Ar2* (3)
Newberry	29.6313, −82.7050	5	*Ne* (5)
Northern Brooksville Ridge (south)	29.11872, −82.44727	1	*NB*
Williston	29.3314, −82.5367	4	*Wl1* (3), *Wl2* (1)
Crescent City		11	5
Pomona Park	29.49041, −81.58434	5	*PP1* (4), *PP2* (1)
Como Lake A	29.46995, −81.60461	5	*Co1* (3), *Co2* (2)
Como Lake B	29.47275, −81.59063	1	*Co3* (1)
DeLand		4	1
DeLand	29.01872, −81.23567		*De* (3)
Cotton Plant		6	3
Morriston A	29.1936, −82.4012	4	*Mo1* (3), *Mo2* (1)
Morriston B	29.1891, −82.3883	2	*Mo3* (2)
Southern Brooksville		15	6
Dunnellon	29.03074, −82.53188	3	*Du1* (2), *Du2* (1)
Weeki Wachee	28.52517, −82.56995	2	*WW* (2)
Weeki Wachee Springs	28.51512, −82.57318	3	*WS* (3)
Withlacoochee State Forest	28.79871, −82.38119	7	*Wi1* (6), *Wi2* (1)
Mount Dora		21	5
Ocala Natl. Forest, Lake Kerr	29.36503, −81.82204	4	*Oc* (4)
Ocala Natl. Forest, firetower	29.17800, −81.77730	6	*Oc* (6)
Ocala Natl. Forest, Astor Park	29.12346, −81.57789	2	*AP* (2)
Ocala Natl. Forest, Tomahawk Lake	29.13381, −81.89107	5	*TL* (5)
Wekiwa Springs State Park	28.71048, −81.46653	4	*We1* (3), *We2* (1)
Lake Apopka	28.54625, −81.70100	2	*LA* (2)
Orlando		3	1
Orlando	28.54230, −81.31950		*Or* (3)
Lake Wales		83	35
Clermont	28.55583, −81.74233	3	*Cl1* (2), *Cl2* (1)
Palatlakaha River Park	28.50334, −81.74950	1	*PR* (1)
Lake Louisa	28.47346, −81.71600	5	*LL1* (3), *LL2* (2)
Lake Marion	28.07444, −81.54659	4	*LM* (4)
Dundee	28.02648, −81.63408	2	*Dd* (2)
Frostproof	27.70455, −81.56120	3	*Fp1* (2), *Fp2* (1)
Snell Creek	28.13136, −81.54161	3	*SC* (3)
Lake Wales	27.92406, −81.60574	5	*LW1* (3), *LW2* (1), *LW3* (1)
Warner College	27.70455, −81.56120	5	*WC* (5)
Hickory Lake Scrub Preserve	27.69647, −81.53964	3	*HL* (1), *Fp2* (1)
Lake Streety	27.69037, −81.56392	4	*LS* (4)
Saddle Blanket Scrub Preserve	27.66950, −81.57633	4	*SB1* (2), *SB2* (2)
Silver Lake	27.56431, −81.52315	5	*SB1* (1), *SL1* (2), *SL2* (1), *SL3* (1)
Carter Creek Scrub Preserve	27.54008, −81.40860	4	*CC* (4)
Highlands Hammock State Park	27.46728, −81.52069	4	*HH* (4)
Sebring	27.44177, −81.41863	6	*Se1* (4), *Se2* (2)
Josephine Creek	27.36860, −81.40173	4	*JC* (4)
Lake Placid	27.24863, −81.30063	5	*LP*1 (1), *LP2* (3), *LP3* (1)
Placid Lakes	27.24169, −81.40142	2	*PL* (2)
Archbold Biological Station	27.18185, −81.35217	8	*ABS1*(5), *ABS2* (1), *ABS3* (2)
Venus	27.10675, −81.33240	1	*Ve* (1)
Winter Haven		8	5
Winter Haven	28.04417, −81.72717	4	*WH1* (2), *WH2* (2)
Bartow	27.96510, −81.77340	2	*Ba* (2)
McLeod Lake	27.96309, −81.74314	2	*Mc1* (1), *Mc2* (1)
Unnamed ridges/uplands			
Dixie	29.60855, −83.07015	5	*Di1* (3), *Di2* (1), *Wl2* (1)
Indian Lake State Forest	29.2835, −82.0539	1	*IL*
Cedar Key	29.18520, −83.01901	4	*CK* (4)
Silver Springs Shores A	29.06142, −81.90823	5	*SS1* (3) *SS2* (2)
Silver Springs Shores B	29.0415, −81.8710	2	*SS3* (2)
Silver Springs Shores C	29.0558, −81.8846	1	*SS4* (1)
Tampa	28.0698, −82.3948	6	*Ta* (6)
