# Supplementary material for: A biogeographical profile of the sand cockroach Arenivaga floridensis and its bearing on origin hypotheses for Florida scrub biota
Source: Ecol Evol. 2018 May 15;8(11):5254–66. doi: 10.1002/ece3.3885 (PMC6010915; doi:10.1002/ece3.3885)
Supplement: Supplementary file 1 [file ECE3-8-5254-s001.docx]

*Ecology and Evolution*

**SUPPORTING INFORMATION**

**A biogeographical profile of the sand cockroach *Arenivaga floridensis* and its bearing on origin hypotheses for Florida scrub biota**

T Lamb, TC Justice, MS Brewer, PE Moler, H Hopkins, & JE Bond

**Supporting Information Figure 1** BI consensus cladogram of the *cox2* dataset showing PP support and tips labeled by *cox2* haplotype. See Appendix for localities.

**Supporting Information Figure 2** BI consensus cladogram of the concatenated dataset showing PP support and tips labeled (using *cox2* haplotype designations). See Appendix for localities.

**Supporting Information Figure 3** Bayesian chronogram for species of *Arenivaga* produced by *BEAST analysis using *cox1* calibration. PP values subtend nodes; purple bars = HPD divergence.

**Supporting Information Figure 4** Bayesian chronogram for the three major lineages of *Arenivaga floridensis* and species of western *Arenivaga* produced by BEAST analysis of the concatenated dataset using *cox1* calibration. All HPD nodes have PP >0.98; purple bars = HPD divergence.
